# Alteration of Average Thickness of Lipid Bilayer by Membrane-Deforming Inclusions

**DOI:** 10.3390/biom13121731

**Published:** 2023-11-30

**Authors:** Oleg V. Kondrashov, Sergey A. Akimov

**Affiliations:** Frumkin Institute of Physical Chemistry and Electrochemistry, Russian Academy of Sciences, 31/4 Leninskiy Prospekt, 119071 Moscow, Russia

**Keywords:** lipid membrane, theory of elasticity, membrane thickness, amphipathic peptide, transmembrane peptide, lipid inclusion, elastic parameters

## Abstract

Thickness of lipid bilayer membranes is a key physical parameter determining membrane permeability and stability with respect to formation of through pores. Most membrane inclusions or impurities like amphipathic peptides, transmembrane peptides, lipid inclusions of a different molecular shape, lipid domains, and protein-lipid domains, locally deform the membrane. The detailed structure of the locally deformed region of the membrane is a kind of “fingerprint” for the inclusion type. However, most experimental methods allow determining only averaged parameters of membranes with incorporated inclusions, thus preventing the direct obtaining of the characteristics of the inclusion. Here we developed a model that allows the obtaining of characteristic parameters of three types of membrane inclusions (amphipathic peptides, transmembrane peptides, monolayer lipid patches) from experimentally observable dependencies of the average thickness of lipid bilayer on the surface concentration of the inclusions. In the case of amphipathic peptides, the model provided the peptide parameters that were in qualitative agreement with the available experimental data.

## 1. Introduction

The lipid bilayer is a main structural element of biological membranes. Membranes are used by cells as weakly permeable, thin envelopes that perform a barrier function. This function allows cells to maintain the composition of cytoplasm, different from the composition of extracellular milieu, and the composition of cell organells different from that of cytoplasm. Membrane stability and, consequently, the efficiency of performing its barrier function strongly depends on membrane thickness: generally, lipid bilayers with smaller thickness are softer and less stable [1,2].

Substances that violate the barrier function of membranes are considered promising antimicrobial agents. These antibiotics include, but are not limited to, amphipathic peptides [3,4,5], membrane-disrupting and penetrating lipocations [6,7], channel-forming peptides [8,9,10,11], polyene antibiotics [12,13]. Amphipathic and channel-forming peptides (like gramicidin A) are definitely known to locally decrease the thickness of lipid bilayers [14,15,16,17], that is observed by means of X-Ray diffraction, molecular dynamics, or inferred from comparison of known bilayer thickness and the length of the membrane-permeating structure formed by the above-mentioned compounds.

Generally, most membrane inclusions or impurities (amphipathic peptides, transmembrane peptides, lipid inclusions of different molecular shape, lipid and protein-lipid domains [18,19], etc.) deform the membrane [20,21]. The lateral length of deformation decay in “conventional” membranes (e.g., formed from dioleoylphosphatidylcholine, DOPC) is of the order of several nanometers [21,22,23]. When the membrane-deforming objects are far-separated, their induced deformations are independent, and their energy is additive. Upon approaching, the deformed zones near the inclusions overlap; the overlapping leads to an effective membrane-mediated lateral interaction [22,23] on the distances of several nanometers, that exceeds typical distances of interactions like screened Debye, van der Waals and dipole–dipole interactions under physiological conditions [24,25,26,27]. The membrane-mediated interaction is significant at large surface concentrations of inclusions. But even in a low concentration, membrane inclusions alter membrane mechanical properties like local bilayer thickness and elastic rigidity [14,15,28,29]. A local disturbance of the lipid bilayer structure contributes to measurable average elastic properties of the membrane that can be observed experimentally. However, the relationship of the local disturbance and the resulting measured deviation of the average characteristics is not straightforward. At the same time, the structure of membrane deformations locally induced by the membrane inclusion depends on the inclusion shape and elastic properties. The establishment of the relationship between nanoscopic, local, and observable average deformations of the membrane may allow extraction of the information about the structure or configuration of the membrane-embedded inclusions. In this present study, we determined how the average membrane thickness changes with the addition of α-helical amphipathic peptides, transmembrane peptides, or a small amount of an impurity lipid differing from the bulk lipid of the membrane.

Spatial distribution of membrane deformations induced by inclusions can be determined in a framework of an adequate theory of elasticity. Such theories formulate possible deformational modes of the considered elastic medium (lipid membrane), and relate deformations and surface density of the elastic energy [20,21,22,23]. The total elastic energy thus becomes a functional on the surface density of the elastic energy. In order to determine the spatial distribution and energy of membrane deformations, one should minimize this functional. A correct minimization problem requires appropriate boundary conditions for the energy functional. As a rule, only lipid deformations are allowed, while membrane-embedded peptides and proteins are deemed absolutely rigid and undeformable. Such rigid membrane inclusions are supposed to impose boundary conditions onto the deformations. In this elastic approach, details of the structure and configuration of the membrane-embedded peptide or protein are completely reflected by the boundary conditions they impose onto the membrane deformations [20,21,22,23], for example, an orientation of lipid molecules and local membrane thickness. Utilizing experimental data on the dependence of membrane thickness on the surface concentration of inclusions, our developed model principally allows the obtaining of boundary conditions imposed by amphipathic peptides and transmembrane peptides. From comparison of calculation results with available experimental data [14,15], we quantitatively determined the boundary director characterizing an average orientation of adjacent lipid molecules for two types (melittin and magainin) of amphipathic peptides incorporated into a lipid monolayer of a membrane.

## 2. Methods

Let us consider a one-component lipid bilayer containing some membrane-deforming inclusions. Such inclusions may be lipid inclusions differing from the main lipid of the membrane; α-helical amphipathic peptide like magainin or melitin; a rigid transmembrane peptide like WALP [30,31]. The ground state of the pure membrane without any inclusions is planar. As the inclusions induce deformations, the ground state of the membrane with even single inclusion would not be a planar configuration [20,21]. Here we determine ground states of lipid membranes with inclusions of different types.

We introduce a Cartesian coordinate system *Oxyz*, the origin of which coincides with the center of the membrane inclusion; the *Oxy* plane is parallel to the plane of the pure membrane in its ground state. The state of the lipid bilayer can be completely characterized by five functions: *H_u_*(*x*, *y*) and *H_l_*(*x*, *y*), which are *z*-coordinates of the upper and lower monolayer neutral surfaces, respectively; *M*(*x*, *y*), which is a *z*-coordinate of the monolayer interface; **n***_u_*(*x*, *y*) and **n***_l_*(*x*, *y*), which are the unit vector fields set on the neutral surfaces of the upper and lower monolayers, respectively. Vectors **n***_u_*(*x*, *y*) and **n***_l_*(*x*, *y*) are called directors, and they characterize an average orientation of lipid hydrophobic chains [32,33]. In the ground state of the pure membrane without inclusions: *H_u_*(*x*, *y*) = *h*, *H_l_*(*x*, *y*) = −*h*, *M*(*x*, *y*) = 0, **n***_u_*(*x*, *y*) = (0, 0, −1) and **n***_l_*(*x*, *y*) = (0, 0, +1), where *h* is the thickness of the hydrophobic part of the lipid monolayer, i.e., the distance from the monolayer interface to the monolayer neutral surface [34].

When the membrane is deformed by a peptide or lipid inclusion, functions *H_u_*(*x*, *y*), *H_l_*(*x*, *y*), *M*(*x*, *y*), **n***_u_*(*x*, *y*), and **n***_l_*(*x*, *y*) generally deviate from constants. When a single inclusion is incorporated into the membrane, the average thickness of the membrane $\langle d_{1}\rangle$ can be determined as follows:

(1) $$\langle d_{1}\rangle =\frac{\int_{S}\left(H_{u}-H_{l}\right)dxdy}{S}=\frac{\int_{S}\left(H_{u}-H_{l}-2h\right)dxdy+2hS}{S}=2h+\frac{\Delta }{S}$$

where *S* is the total area of the membrane; $\Delta =\int_{S}\left(H_{u}-H_{l}-2h\right)dxdy$; the integration is performed over the membrane area *S*. The integral Δ is accumulated only in the vicinity of membrane inclusions, because the value of (*H_u_* − *H_l_* − 2*h*) generally decays exponentially with increasing distance from the inclusion [20,21,22,23].

In the case of *N* identical inclusions incorporated into the membrane in a low surface concentration *C*, we can neglect lateral interactions between the inclusions and write down the following linear expression for the average thickness of the membrane 〈d〉:

(2) $$\langle d\rangle =2h+\frac{N\Delta }{S}=2h+C\Delta .$$

So, in a low concentration limit the average membrane thickness is completely characterized by the concentration of inclusions and the thickness disturbance Δ induced by single inclusion.

In order to calculate Δ for several types of membrane inclusions, we utilized the theory of elasticity of lipid membranes originally developed by Hamm and Kozlov [33] and further generalized in the works [21,22,23] to include additional deformational modes. The corresponding functional of the monolayer elastic energy:

(3) $$\begin{array}{l} W=\int_{S_{m}}dS\{\frac{B}{2}{\left[\mathrm{div}n+J_{0}\right]}^{2}-\frac{B}{2}J_{0}^{2}+\frac{K_{t}}{2}t^{2}+\frac{\sigma }{2}{\left[grad\;H\right]}^{2}+\\ +\frac{K_{a}}{2}\alpha^{2}+K_{G}K+\frac{K_{rot}}{2}{\left[rot\;n\right]}^{2}\} \end{array}$$

accounts for the deformations of splay (the first and second terms), tilt (the third term), lateral tension (the fourth term), lateral compression (the fifth term), Gaussian splay (the sixth term), and twist (the last term), respectively; the integration is performed over the neutral surface of the lipid monolayer *S_m_*. The energy functional (3) is quadratic in deformations, and it is valid for small deformations only; in particular, within the required accuracy, **n***_l_*_,*u*_ = (*n_x_*(*x*, *y*), *n_y_*(*x*, *y*), ±1). Here, *B*, *K_t_*, *K_a_*, *K_G_*, and *K_rot_* are elastic moduli (per monolayer) of splay, tilt, lateral compression, Gaussian splay, and twist, respectively; *J*_0_ is the spontaneous curvature of the monolayer; *σ* is the monolayer lateral tension; *α* is the relative change of the area per lipid molecule at the monolayer neutral surface; $K=\frac{\partial n_{x}}{\partial x}\frac{\partial n_{y}}{\partial y}-\frac{\partial n_{x}}{\partial y}\frac{\partial n_{y}}{\partial x}$ is the Gaussian splay; $\mathrm{div}n=\frac{\partial n_{x}}{\partial x}+\frac{\partial n_{y}}{\partial y}$ is the divergence of the director along the neutral surface; $rot\;n={\left(0,\;0,\;\frac{\partial n_{y}}{\partial x}-\frac{\partial n_{x}}{\partial y}\right)}^{T}$ is the vector of rotor of the director (*T* is transposition); $grad\;H={\left(\frac{\partial H}{\partial x},\;\frac{\partial H}{\partial y},\;0\right)}^{T}$ is the vector of gradient of *H*(*x*, *y*). Taking into account that the bulk modulus of membranes is very high [35], we assume that the hydrophobic part of a lipid monolayer is locally volumetrically incompressible, i.e., the volume of its any small element does not change upon deformations. This condition imposes a constraint on the deformation fields. Within this condition, the elastic energy functional (3) for the bilayer membrane can be rewritten as:

(4) $$\begin{array}{l} W=\int_{S_{u}}dS_{u}(\frac{B}{2}{\left(\mathrm{div}n_{u}+J_{u}\right)}^{2}-\frac{B}{2}J_{u}^{2}+\frac{K_{t}}{2}{\left(n_{u}-grad\;H_{u}\right)}^{2}+\frac{\sigma }{2}{\left(grad\;H_{u}\right)}^{2}+\\ +\frac{K_{a}}{2}\frac{1}{h^{2}}{\left(h-\frac{h^{2}}{2}\mathrm{div}n_{u}+M-H_{u}\right)}^{2}+K_{G}K_{u}+\frac{K_{rot}}{2}{\left(rot\;n_{u}\right)}^{2})+\\ +\int_{S_{l}}dS_{l}(\frac{B}{2}{\left(\mathrm{div}n_{l}+J_{l}\right)}^{2}-\frac{B}{2}J_{l}^{2}+\frac{K_{t}}{2}{\left(n_{l}+grad\;H_{l}\right)}^{2}+\frac{\sigma }{2}{\left(grad\;H_{l}\right)}^{2}+\\ +\frac{K_{a}}{2}\frac{1}{h^{2}}{\left(h-\frac{h^{2}}{2}\mathrm{div}n_{l}-M+H_{l}\right)}^{2}+K_{G}K_{l}+\frac{K_{rot}}{2}{\left(rot\;n_{l}\right)}^{2}), \end{array}$$

where the indices “*u*” and “*l*” correspond to the upper and lower monolayers, respectively; the integration is performed over the neutral surfaces of the respective monolayers.

All deformations should be limited everywhere, and should vanish at large distances from the inclusion, i.e.,

(5) $$\begin{array}{l} H_{u}\left(\infty \right)=h,\;H_{l}\left(\infty \right)=-h,\;M\left(\infty \right)=0,\\ n_{u}\left(\infty \right)=(0,\;0,-1),\;n_{l}\left(\infty \right)=(0,\;0,+1). \end{array}$$

Depending on the type of the membrane inclusions, they impose different boundary conditions on the elastic energy functional (4). Below, we explicitly consider boundary conditions imposed onto the deformation fields by amphipathic peptides, transmembrane peptides and lipid inclusions.

### 2.1. Amphipathic Peptide

**Figure 1:**
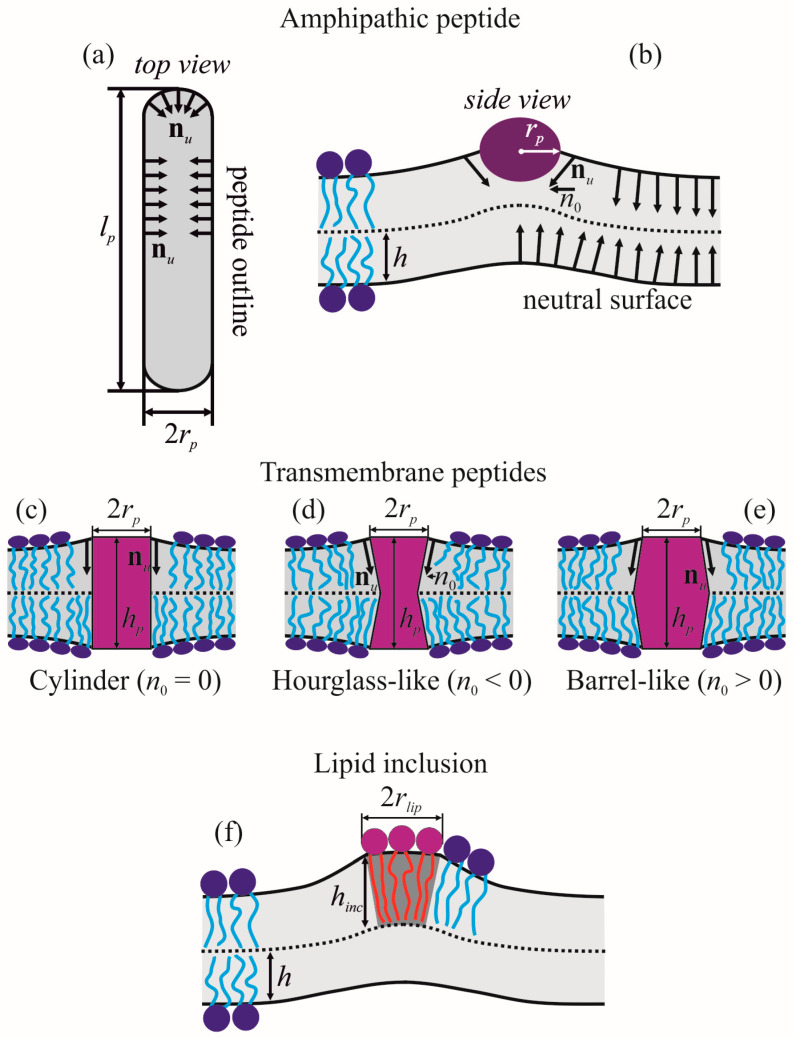
Schematic representation of boundary conditions imposed by amphipathic peptide, transmembrane peptides and lipid inclusion. (**a**) amphipathic peptide, top view; (**b**) amphipathic peptide, side view along the longitudinal axis of the peptide α-helix; (**c**) cylindrical transmembrane peptide (*n*_0_ = 0); (**d**) hourglass-like transmembrane peptide (*n*_0_ < 0); (**e**) barrel-like transmembrane peptide (*n*_0_ > 0); (**f**) lipid inclusion.

For definiteness, we considered α-helical amphipathic peptides. Such peptides consist of hydrophobic and polar (or charged) amino acids combined in such a way that one side-surface of α-helix is hydrophobic, while the opposite side-surface is polar. In order to hide their hydrophobic surfaces from contact with water, amphipathic peptides partially incorporate into the membrane monolayer. The incorporation requires a lateral shifting of polar lipid heads away, resulting in a jump of lipid orientation on the left and right boundaries of the amphipathic peptide. This effect can be formally expressed as the following boundary conditions imposed on the director field [23,36] at the peptide outline Γ at the neutral surface of the upper monolayer (Figure 1a):

(6) $$n_{n,u}(\Gamma )=n_{0},\;n_{\tau ,u}(\Gamma )=0,$$

where **n***_n_*_,*u*_ and **n***_τ_*_,*u*_ are, respectively, the normal and tangential to the contour Γ components of the director field **n***_u_* of the upper monolayer. The exact value of *n*_0_ is unknown; it depends on the details of peptide structure and lipid-peptide intermolecular interactions. However, based on geometric meaning of the director, *n*_0_ can be estimated as (Figure 1b) [23,36]:

(7) $$\left|n_{0}\right|=\frac{r_{p}}{\sqrt{r_{p}^{2}+h^{2}}},$$

where *r_p_* is the radius of α-helix. The boundary director is directed beneath the incorporated amphipathic peptide (Figure 1b). Of note, as the elastic energy functional (4) is quadratic in deformations, the value of Δ should be linear on *n*_0_: Δ = *ηn*_0_, where *η* is some constant independent on *n*_0_. The same should hold for amplitudes of all deformation fields in (4).

### 2.2. Transmembrane Peptide

Transmembrane peptide incorporated into a lipid bilayer imposes boundary conditions both on the director field and membrane thickness at the peptide boundary outlines at the neutral surfaces of the upper and lower monolayers [22,37]:

(8) $$\begin{array}{l} n_{n,u}(\Gamma )=n_{0u},\;n_{\tau ,u}(\Gamma )=0,\\ n_{n,l}(\Gamma )=n_{0l},\;n_{\tau ,u}(\Gamma )=0,\\ H_{u}(\Gamma )-H_{l}(\Gamma )=h_{p}, \end{array}$$

where *h_p_* is the length of transmembrane part of the peptide; *n*_0*u*_, *n*_0*l*_ are projection of boundary directors onto *Oxy* plane (Figure 1c–e). We assumed that outlines of the peptide boundary on the neutral surfaces of the upper (Γ*_u_*) and lower (Γ*_l_*) monolayers are identical circles of the radius *r_p_* and their center coordinates are (*x*, *y*) = (0, 0); thus, Γ*_u_* = Γ*_l_* = Γ. For definiteness, we considered the case of symmetric transmembrane peptides, *n*_0*u*_ = *n*_0*l*_ = *n*_0_. Negative *n*_0_ corresponds to the hourglass-like shape of the peptide (Figure 1d), positive *n*_0_—to barrel-like shape (Figure 1e), and *n*_0_ = 0—to cylindrical peptides (Figure 1c) [37]. For a quadratic energy functional (4), Δ should linearly depend on *n*_0_ and (*h_p_* − 2*h*), i.e., it can be expressed in the form Δ = *βn*_0_ + *γ*(*h_p_* − 2*h*), where *β*, *γ* are constants that do not depend on *n*_0_ and *h_p_*.

### 2.3. Lipid Inclusion

Lipid inclusions do not impose any specific boundary conditions, except continuity of the neutral surface and director (Figure 1f). Unlike undeformable peptide inclusions, lipid inclusions can be deformed and the energy of their deformation should be calculated in the framework of the same elastic theory as that of the surrounding membrane, e.g., utilizing the elastic energy functional (4). However, generally, the elastic parameters of lipid inclusions differ from those of the membrane, and this difference should be taken into account. As a lipid inclusion, we considered a circular patch of lipid monolayer of the radius *r_lip_*, the center of which has coordinates (*x*, *y*) = (0, 0). The patch is located in the upper monolayer of the membrane (Figure 1f).

We can determine how Δ depends on spontaneous curvature of lipid inclusion *J*_0*inc*_. If we fix the value of the radial component of the director at the inclusion boundary, **n***_r_*(*r_lip_*) = *n*, and the thickness of the bilayer at the inclusion boundary [*H_u_*(*r_lip_*) − *H_l_*(*r_lip_*)] = *d*, we can write down the following expression for the elastic energy *W*:

(9) $$\begin{array}{l} W=\left[\alpha_{1}n^{2}+\beta_{1}{\left(d-h_{inc}-h\right)}^{2}+\gamma_{1}n(d-h_{inc}-h)+2\pi r_{lip}BJ_{0}n\right]+\\ +\left[\alpha_{2}n^{2}+\beta_{2}{\left(d-2h\right)}^{2}+\gamma_{1}n(d-2h)-2\pi r_{lip}B_{inc}J_{0inc}n\right], \end{array}$$

where *h_inc_* is the thickness of the lipid inclusion monolayer; *α*_1,2_, *β*_1,2_, *γ*_1,2_ are constants depending on elastic parameters of the bulk lipid and the lipid of the inclusion except spontaneous curvature *J*_0*inc*_ and thickness *h_inc_*. The first and second square brackets correspond to energies of membrane deformations in the circle of the radius *r_lip_* and in the outer region, respectively. Minimizing (9) with respect to *n* and *d*, we obtain what *n* ∝ (*kJ*_0*inc*_ + *b*), where *k* and *b* are constants that do not depend on *J*_0*inc*_. As the elastic energy functional (4) is quadratic on deformations, we can conclude that Δ = (*kJ*_0*inc*_ + *b*).

In order to obtain the values of the parameters *η*, *β*, *γ*, *k*, *b*, we numerically minimized the elastic energy functional (4) using the finite elements method, essentially as described in details in the works [22,23,36,37]. The minimization routine was written in Mathematica 11.3 (Wolfram Research, Champaign, IL, USA) using its standard functions; the corresponding program can be obtained from the authors by request.

### 2.4. Parameters

To obtain quantitative results, the following parameters were used. For DOPC, which was considered as the main lipid component of the membrane: *B* = 10 *k_B_T* [2], *K_t_* = 40 mN/m [33], *K_a_* = 133 mN/m [2], *σ* = 0.1 mN/m, *K_rot_* = *B*/2 = 5 *k_B_T*, *K_G_* = −*B*/2 = −5 *k_B_T* [23,36,37,38], *J*_0_ = −0.091 nm^−1^ [39], *h* = 1.45 nm [2,34]. In the work [14], the average thickness of the membrane made from palmitoyloleoylphosphatidylcholine (POPC):palmitoyloleoylphosphatidylserine (POPS) 3:1 was measured as a function of the mole fraction of adsorbed amphipathic peptide melittin. For this lipid mixture, we utilized the following parameters: *B* = 11 *k_B_T* [2], *K_t_* = 40 mN/m [33], *K_a_* = 117 mN/m [2], *σ* = 0.1 mN/m, *K_rot_* = *B*/2 = 5.5 *k_B_T*, *K_G_* = −*B*/2 = −5.5 *k_B_T* [23,36,37,38], *J*_0_ = 0 [39], *h* = 1.46 nm [2,34]. For the amphipathic peptide: *l_p_* = 5 nm, *r_p_* = 0.65 nm. For the transmembrane peptide: *r_p_* = 0.65 nm. For the lipid inclusion: *r_lip_* = 0.5 nm or *r_lip_* = 0.662 nm in the case of thin lipid inclusion, like photoswitchable lipid OptoDArG, *K_t_* = 40 mN/m [33], *K_a_* = 133 mN/m, *σ* = 0.1 mN/m, *K_rot_* = *B*/2 = 5 *k_B_T*, *K_G_* = −*B*/2 = −5 *k_B_T*.

## 3. Results

### 3.1. Amphipathic Peptide

**Figure 2:**
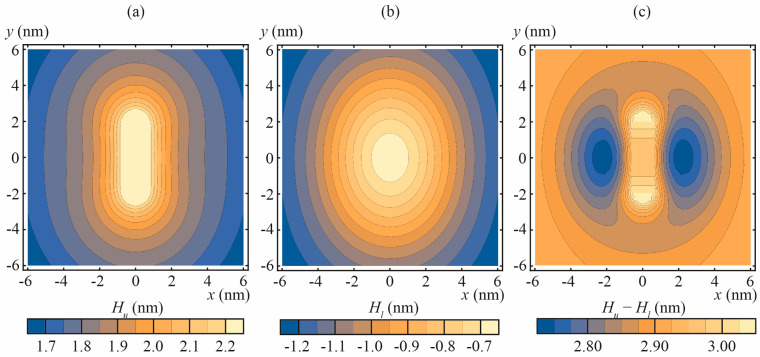
The shapes of the neutral surfaces of the upper and lower monolayers and the thickness of the DOPC membrane with incorporated amphipathic peptide imposing the normal projection of the boundary director |*n*_0_| = 0.4. (**a**) the shape of the neutral surface of the upper monolayer, *H_u_*(*x*, *y*); (**b**) the shape of the neutral surface of the lower monolayer, *H_l_*(*x*, *y*); (**c**) the thickness of the membrane, [*H_u_*(*x*, *y*) − *H_l_*(*x*, *y*)].

Numerically calculated shapes *H_u_*(*x*, *y*), *H_l_*(*x*, *y*) of neutral surfaces of the upper and lower monolayers of DOPC membrane with incorporated amphipathic peptide are illustrated in Figure 2a,b for particular case of *n*_0_ = −0.4, as derived from Equation (7) for *r_p_* = 0.65 nm, *h* = 1.45 nm. The membrane thickness [*H_u_*(*x*, *y*) − *H_l_*(*x*, *y*)] is presented in Figure 2c. The obtained dependence of Δ on *n*_0_ for DOPC membrane is: Δ = 9.16 × *n*_0_ (in the units of nm^3^); for POPC:POPS 3:1 membrane: Δ = 9.36 × *n*_0_ (in the units of nm^3^). The similarity of the proportionality coefficient between Δ and *n*_0_ for DOPC and POPC:POPS membrane is not surprising, as the elastic parameters of these lipids differ, but slightly.

### 3.2. Transmembrane Peptide

**Figure 3:**
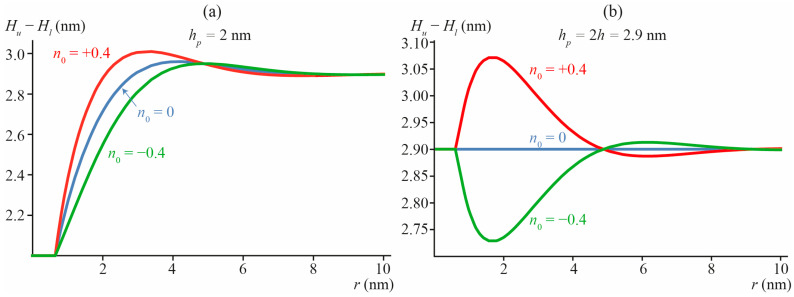
The thickness of the DOPC membrane [*H_u_*(*x*, *y*) − *H_l_*(*x*, *y*)] with incorporated transmembrane peptide of the length: (**a**) *h_p_* = 2 nm; (**b**) *h_p_* = 2*h* = 2.9 nm (no mismatch with the surrounding membrane), imposing the boundary director: *n*_0_ = −0.4 (hourglass-like peptide, green curves), 0 (cylindrical peptide, blue curves), +0.4 (barrel-like peptide, red curves). The radial coordinate *r* is the distance to the center of the peptide: *r* = (*x*^2^ + *y*^2^)^1/2^.

Radial distribution of the thickness of the DOPC membrane with incorporated transmembrane peptide is shown in Figure 3 for different values of the transmembrane peptide length and boundary director. Due to the symmetry with respect to the monolayer interface, [*H_u_*(*x*, *y*) − *H_l_*(*x*, *y*)] = 2*H_u_*(*x*, *y*) = −2*H_l_*(*x*, *y*). The determined dependence of Δ on *n*_0_ and *h_p_* is: Δ = 10.72 × *n*_0_ + 3.96 × (*h_p_* − 2*h*) (in the units of nm^3^).

Figure 3a illustrates a deviation of the membrane thickness in the vicinity of short transmembrane peptide, the length *h_p_* = 2 nm of which is smaller than the bilayer equilibrium thickness 2*h* = 2.9 nm. In Figure 3b, the case of no mismatch between the transmembrane peptide length and bilayer thickness (*h_p_* = 2*h* = 2.9 nm) is illustrated. From Figure 3b, it follows that even when *h_p_* = 2*h*, but *n*_0_ ≠ 0 (red and green curves in Figure 3b), deformations of the membrane arise leading to an alteration of the local thickness of the bilayer.

### 3.3. Lipid Inclusion

We considered two types of lipid inclusions. The first type is “common” lipid inclusion with splay modulus ranging from 5 to 20 *k_B_T*, the monolayer thickness *h_inc_* of which varies from 1.2 to 1.7 nm and spontaneous curvature ranges from −0.6 to +0.2 nm^−1^. This type corresponds to “common” lipids like DOPC, POPC, etc. In addition, we especially considered the second type of so-called “thin” lipid inclusions with *h_inc_* = 0.7 nm. This type models photoswitchable lipids having cis-trans isomerizable diazobenzene group in one or two hydrophobic chains, like PhoDAG and OptoDArG [40,41,42], respectively. In cis-conformation such lipids are characterized by a small monolayer thickness and strongly negative spontaneous curvature [43], while in trans-conformation these lipids can be considered as “common” ones. Photoswitchable lipids are a convenient biophysical tool, although their physical parameters are not characterized well to date.

**Figure 4:**
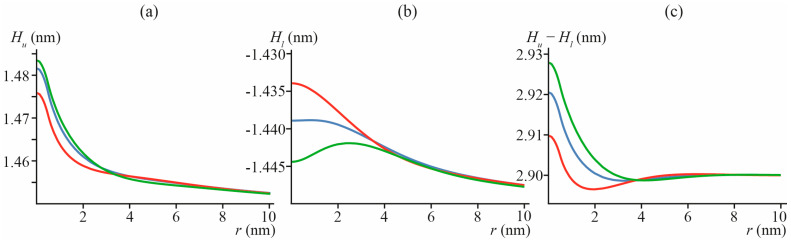
Shapes of neutral surfaces of membrane monolayers, (**a**) *H_u_*(*x*, *y*), and (**b**) *H_l_*(*x*, *y*), and membrane thickness (**c**) [*H_u_*(*x*, *y*) − *H_l_*(*x*, *y*)], in particular case of *B_imp_* = 10 *k_B_T*, *J*_0*inc*_ = 0 ≠ *J*_0_ = −0.091 nm^−1^, *h_inc_* = *h* = 1.45 nm (blue), *h_inc_* = 1.35 nm (red) and *h_inc_* = 1.55 nm (green), as a function of distance to lipid inclusion center *r* = (*x*^2^ + *y*^2^)^1/2^.

Calculated shapes of membrane neutral surfaces, *H_u_*(*x*, *y*) and *H_l_*(*x*, *y*), and membrane thickness [*H_u_*(*x*, *y*) − *H_l_*(*x*, *y*)] are presented in Figure 4 for particular cases of “common” lipid inclusion with *B_inc_* = 10 *k_B_T*, *J*_0*inc*_ = 0, and *h_inc_* = *h*, *h +* 0.1 nm, *h* − 0.1 nm. From Figure 4c, it follows that even in the case of no thickness mismatch, *h_inc_* = *h*, the membrane thickness [*H_u_*(*x*, *y*) − *H_l_*(*x*, *y*)] can still locally deviate from 2*h* if *J*_0*inc*_ ≠ *J*_0_ (see blue curve in Figure 4c). In addition, a difference in the spontaneous curvatures of the bulk lipid and lipid inclusion can lead to a local increase in the membrane thickness even in the case of *h_inc_* < *h* (see red curve in Figure 4c). Of note, the typical length of bilayer thickness relaxation is of the order of 3–4 nm (Figure 4c).

**Figure 5:**
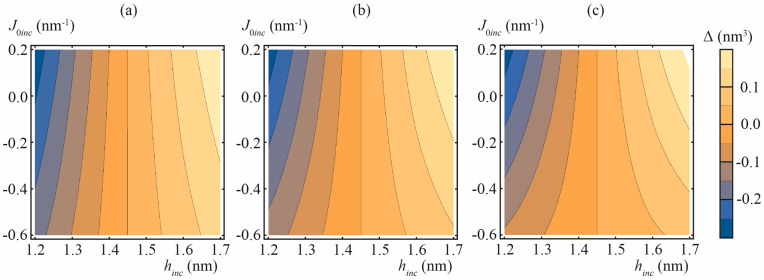
Calculated dependencies of Δ on thickness *h_inc_* and spontaneous curvature *J*_0*inc*_ of “common” lipid inclusion. (**a**) *B_inc_* = 5 *k_B_T*; (**b**) *B_inc_* = 10 *k_B_T*; (**c**) *B_inc_* = 20 *k_B_T*.

In Figure 5, we presented the calculated values of Δ for “common” lipid inclusion with splay modulus *B_inc_* = 5, 10, and 20 *k_B_T*, spontaneous curvature *J*_0*inc*_ varying from −0.6 to 0.2 nm^−1^ and thickness of the inclusion *h_inc_* varying from 1.2 to 1.7 nm. Of note, in the case of zero mismatch, *h_inc_* = *h*, the average thickness of the membrane is strictly 2*h* (i.e., Δ = 0) independently on *B_inc_* and *J*_0*inc*_ values, even though the deformations do arise and membrane local thickness deviates from 2*h* (c.f. blue curve in Figure 4c).

**Figure 6:**
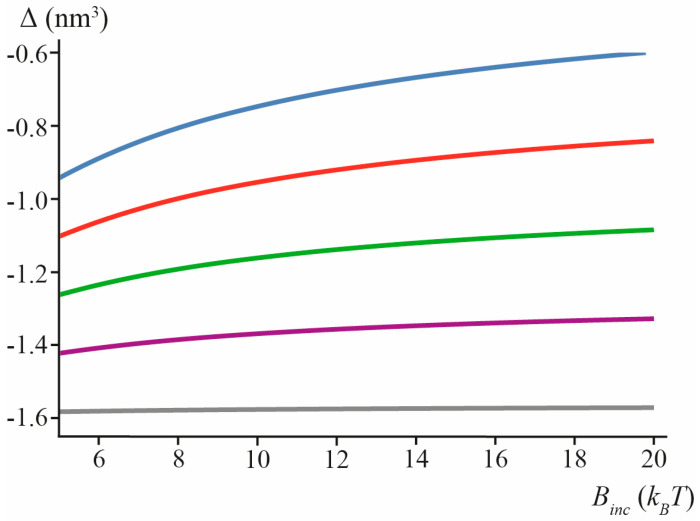
Calculated values of Δ in the case of “thin” lipid inclusion (*h_inc_* = 0.7 nm) for *J*_0*inc*_ = −0.6 nm^−1^ (blue), *J*_0*inc*_ = −0.4 nm^−1^ (red), *J*_0*inc*_ = −0.2 nm^−1^ (green), *J*_0*inc*_ = 0 (purple), *J*_0*inc*_ = +0.2 nm^−1^ (gray) as a function of inclusion splay modulus, *B_inc_*.

The calculated values of Δ for “thin” lipid inclusion (*h_inc_* = 0.7 nm) are presented in Figure 6 for different spontaneous curvatures and splay moduli of the inclusion.

## 4. Discussion

Most membrane inclusions cause elastic deformations of lipid bilayers. A detailed structure of the locally deformed region of the membrane is a kind of “fingerprint” for the inclusion type. However, most experimental methods allow determining only averaged parameters of membranes with incorporated inclusions, thus preventing from direct obtaining of the characteristics of the inclusion. A derivation of the structural information from the measured average parameters is an inverse problem, the solution of which is always model-dependent. Molecular dynamics (MD) allows determining configurations of membrane-embedded inclusions within an atomistic resolution. However, structural information obtained from MD may be too detailed: it is preferable to characterize membrane inclusions by a few number of effective parameters, while MD provides coordinates of all atoms of the modeled system, i.e., enormous number of the parameters. The data of MD should thus be somehow averaged to converge to one or several effective characteristics of the system. However, the averaging may yield the parameter differing from those obtained from physical experiments [17] or from the theory of elasticity [44,45].

In the present work, we developed a model that allows solving the inverse problem: deriving one to several effective elastic parameters from the measured average thickness of the membrane for three types of membrane-deforming inclusions: amphipathic peptides, transmembrane peptides, lipid inclusions. In the framework of the model, amphipathic peptides of equal length are characterized by the only parameter, the normal projection of the boundary director, *n*_0_. Dependencies of the average thickness of the membrane on the mole fraction of incorporated amphipathic peptides were determined in works [14,15] for melittin in DOPC membrane [14] and magainin in POPC:POPS 3:1 membrane [15]. For mole fractions smaller than about 1/65 peptide/lipid, the dependencies are linear, and thus they can be readily fitted by the linear relation Δ = *ηn*_0_ where the only fitting parameter is *n*_0_ (Δ is measured in [14,15]; *η* was calculated here). This yields *n*_0_ = −0.42 for melittin and *n*_0_ = −0.48 for magainin. Of note, the estimation of the boundary director based on its oversimplified geometric interpretation, Equation (7), yields *n*_0_ = −0.4 that is surprisingly close to the determined value for melittin. In our recent work on membrane-mediated lateral interactions of amphipathic peptides, we used the same value of the boundary director [46]. This difference in the projections of the boundary directors is in a qualitative agreement with available data on magainin and melittin structures. When incorporated into a membrane, the longitudinal axis of magainin α-helix is about parallel to the membrane plane, and the molecule can be approximately considered as a cylinder [47,48]. Melittin has a relatively flexible loop in its structure, and can be approximately considered as two α-helical non-equal parts connected by this loop [48,49]. The smaller α-helical part of melittin is more hydrophobic than the larger one, and this part is incorporated deeper into the membrane [48,49], that should result in smaller (by the absolute value) projection of the boundary director *n*_0_; this agrees qualitatively with the predictions of our model. Magainin and melittin are known as classical antimicrobial peptides that effectively disrupt membranes by forming through pores in them [3,50,51,52,53,54]. The projection of the boundary director *n*_0_ is a key parameter determining the energy landscape of pore formation: the larger the |*n*_0_|, the higher the poration efficiency should be [55].

The model developed here can be utilized to retrieve characteristic parameters of the considered types of membrane inclusions. This requires measurements of dependencies of the average membrane thickness on the inclusion surface concentration in a low concentration (linear) regime. As transmembrane peptides and lipid inclusions are each described by two effective parameters (*n*_0_ and *h_p_*, *J*_0*inc*_ and *h_inc_*, respectively) their determination requires at least two dependencies of the average membrane thickness on the inclusion surface concentration, measured in two different membranes. Unfortunately, to date, the measurements were not performed for transmembrane peptides, lipid inclusions, or photoswitchable lipids (as far as we know).

Gramicidin A is a peptide, the transmembrane dimers of which are ion-conducting channels [9]. The length of the dimer may differ from the membrane thickness that leads to membrane deformations in the vicinity of both gramicidin monomers and dimers [10,16,17,21,23]. The deformations contribute to the energy of monomeric and dimeric states, and the channel characteristics depend on the elastic properties of the membrane [10,23]. For this reason, gramicidin is considered a molecular force sensor [10]. To properly describe gramicidin based on the theory of elasticity of membranes, one should know boundary conditions imposed by gramicidin monomer and dimers. The boundary conditions can principally be determined from the model developed here. The change of lipid bilayer thickness upon incorporation of gramicidin was measured in the work [56]. It was shown that gramicidin induces thinning of dimiristoylphosphatidylcholine (DMPS) and thickening of dilauroylphosphatidylcholine (DLPC) membranes. However, the peptide/lipid ratio in these experiments was very high, 1/10 peptide/lipid. This corresponds to only about 2.5 nm gramicidin–gramicidin separations, meaning that deformations induced by gramicidin molecules should definitely overlap. Besides, the deformations induce by gramicidin dimers are generally different from those induced by monomers. Thus, there are two types of membrane-deforming inclusions in such the system: gramicidin monomers and dimers. These inclusions are in equilibrium, and the equilibrium surface concentration of dimers depends on elastic parameters of the membrane. In principle, our elastic model can be generalized to describe this case of non-ideal mixture of gramicidin monomers and dimers, where all molecular species laterally interact with each other; the necessary theoretical background was partially developed in our recent work [23]. However, the experiment of the work [56] provides only one measured value—the membrane thickness—at single concentration of gramicidin. From this value, it is impossible to determine the constant of equilibrium and two sets of boundary conditions imposed by monomers and dimers onto the membrane; additional experimental data are required.

Recently, the energy of transition of single gramicidin dimer from DLPC to DMPC membrane was determined as 3.7 *k_B_T* from molecular dynamics simulations [17]. In this work, the dimer is characterized by the only parameter, its length *h_p_*. However, the length of gramicidin dimer was determined experimentally as *h_p_* = 2.2 nm, independently on the membrane composition [56]. In the oversimplified model, where gramicidin dimer is characterized by *h_p_* only, it is impossible to obtain the measured value of the transition energy using the value *h_p_* = 2.2 nm. In the work [17], it was supposed that the effective “deformational” dimer length should be larger than its actual length by 0.4 nm. However, if transmembrane dimer of gramicidin is characterized by two parameters, *n*_0_ and *h_p_*, one can estimate the value *n*_0_ = −0.45 [37] from the measured transition energy [17], using the actual length of the dimer *h_p_* = 2.2 nm. Thus, physical experiments and molecular dynamics modeling can be combined to derive boundary conditions imposed by various membrane-deforming inclusions.

## Data Availability

No new data were created or analyzed in this study. Data sharing is not applicable to this article.
